# Supplementary material for: Psychological and financial impacts of COVID-19-related travel measures: An international cross-sectional study
Source: PLoS One. 2022 Aug 5;17(8):e0271894. doi: 10.1371/journal.pone.0271894 (PMC9355190; doi:10.1371/journal.pone.0271894)
Supplement: S1 Table — (DOCX) [file pone.0271894.s001.docx]

| **Supplementary file 1.** Associations and variance between participant factors and levels of depression, anxiety, and stress (n=1363) | | | | | | | | |
| --- | --- | --- | --- | --- | --- | --- | --- | --- |
|  | **Depression** | | | | | **χ 2** | **df** | ***p*** |
| **Variables** | **Normal**  **n (%)** | **Mild**  **n (%)** | **Moderate**  **n (%)** | **Severe**  **n (%)** | **Extremely Severe**  **n (%)** |  |  |  |
| **Eligibility** |  |  |  |  |  | 37.856 | 8 | **<.001** |
| Separated from partner/spouse | 41 (9.6) | 35 (8.2) | 76 (17.8) | 66 (15.5) | 209 (48.9) |  |  |  |
| Separated from immediate family | 105 (15.2) | 77 (11.2) | 177 (25.7) | 91 (13.2) | 240 (34.8) |  |  |  |
| Temporary visa holders | 41 (16.7) | 18 (7.3) | 41 (16.7) | 40 (16.3) | 106 (43.1) |  |  |  |
| **Gender (n=1350)** |  |  |  |  |  | 14.745 | 4 | **.005** |
| Female | 119 (11.9) | 101 (10.1) | 227 (22.7) | 139 (13.9) | 416 (41.5) |  |  |  |
| Male | 67 (19.3) | 29 (8.3) | 65 (18.7) | 56 (16.1) | 131 (37.6) |  |  |  |
| **Mental health services offered** |  |  |  |  |  | 15.561 | 4 | **.004** |
| Yes | 20 (7.5) | 21 (7.9) | 54 (20.3) | 43 (16.2) | 128 (48.1) |  |  |  |
| No | 167 (15.2) | 109 (9.9) | 240 (21.9) | 154 (14) | 427 (38.9) |  |  |  |
| **Medical advice offered** |  |  |  |  |  | 22.041 | 4 | **<.001** |
| Yes | 14 (7.2) | 17 (8.7) | 31 (15.9) | 26 (13.3) | 107 (54.9) |  |  |  |
| No | 173 (14.8) | 113 (9.7) | 263 (22.5) | 171 (14.6) | 448 (38.4) |  |  |  |
| **Government financial assistance offered** |  |  |  |  |  | 6.352 | 4 | **.174** |
| Yes | 12 (9.7) | 11 (8.9) | 24 (19.4) | 14 (11.3) | 63 (50.8) |  |  |  |
| No | 175 (14.1) | 119 (9.6) | 270 (21.8) | 183 (14.8) | 492 (39.7) |  |  |  |
| **Traveler registration offered** |  |  |  |  |  | 6.304 | 4 | **.178** |
| Yes | 33 (14.3) | 29 (12.6) | 57 (24.7) | 28 (12.1) | 84 (36.4) |  |  |  |
| No | 154 (13.6) | 101 (8.9) | 237 (20.9) | 169 (14.9) | 471 (41.6) |  |  |  |
| **Emergency accommodation offered** |  |  |  |  |  | 2.270 | 4 | .693^a^ |
| Yes | 2 (10.5) | 3 (15.8) | 4 (21.1) | 1 (5.3) | 9 (47.4) |  |  |  |
| No | 185 (13.8) | 127 (9.4) | 290 (21.6) | 197 (14.5) | 555 (40.7) |  |  |  |
| **Financial stress** |  |  |  |  |  | 54.377 | 4 | **<.001** |
| Yes | 104 (10.7) | 89 (9.2) | 190 (19.6) | 139 (14.3) | 449 (46.2) |  |  |  |
| No | 83 (21.2) | 41 (10.5) | 104 (26.5) | 58 (14.8) | 106 (27) |  |  |  |
| **Homelessness** |  |  |  |  |  | 18.707 | 4 | **<.001** |
| Yes | 13 (10.2) | 11 (8.6) | 21 (16.4) | 9 (7) | 74 (57.8) |  |  |  |
| No | 174 (14.1) | 119 (9.6) | 273 (22.1) | 188 (15.2) | 481 (38.9) |  |  |  |
| **Employment change** |  |  |  |  |  | 20.450 | 4 | **<.001** |
| Yes | 92 (13.1) | 67 (9.6) | 121 (17.3) | 102 (14.6) | 318 (45.4) |  |  |  |
| No | 95 (14.3) | 63 (9.5) | 173 (26.1) | 95 (14.3) | 237 (35.7) |  |  |  |
| **Children** |  |  |  |  |  | 35.878 | 4 | **<.001** |
| Yes | 101 (18.7) | 59 (10.9) | 124 (23) | 83 (15.4) | 173 (32) |  |  |  |
| No | 86 (10.4) | 71 (8.6) | 170 (20.7) | 114 (13.9) | 382 (46.4) |  |  |  |
| **Employment during COVID-19** |  |  |  |  |  | 39.935 | 24 | **.044** |
| Healthcare worker | 21 (15.6) | 17 (12.6) | 38 (20.7) | 26 (19.3) | 42 (31.9) |  |  |  |
| Government worker | 6 (9) | 5 (7.5) | 16 (23.9) | 17 (25.4) | 23 (34.3) |  |  |  |
| Essential services worker | 23 (13) | 11 (6.2) | 30 (16.9) | 25 (14.1) | 88 (49.7) |  |  |  |
| Educator | 17 (12.7) | 16 (11.9) | 36 (26.9) | 17 (12.7) | 48 (35.8) |  |  |  |
| Other worker | 88 (14.1) | 64 (10.2) | 139 (22.2) | 87 (13.9) | 249 (39.6) |  |  |  |
| Not in paid work | 25 (13) | 15 (7.8) | 37 (19.2) | 20 (10.4) | 96 (49.7) |  |  |  |
| Retired | 7 (22.6) | 2 (6.5) | 8 (25.8) | 5 (16.1) | 9 (29) |  |  |  |
| **Chronic Illness** |  |  |  |  |  | 16.092 | 8 | **.041** |
| Yes | 9 (6.1) | 14 (9.5) | 27 (18.4) | 28 (19) | 69 (46.9) |  |  |  |
| No | 174 (14.8) | 111 (9.5) | 259 (22.1) | 159 (13.5) | 471 (40.1) |  |  |  |
| Unsure | 4 (9.5) | 5 (11.9) | 8 (19) | 10 (23.8) | 15 (35.7) |  |  |  |
|  | | | | | | **F** | **df** | ***p*** |
| **Expenditure** |  |  |  |  |  | 1.458 | 4, 1358 | .213 |
| **Age** |  |  |  |  |  | 8.185 | 4, 1368 | **<.001** |
| **Time separated or awaiting immigration** |  |  |  |  |  | .971 | 4, 1358 | .422 |
|  | **Anxiety** | | | | | **χ 2** | **df** | ***p*** |
| **Eligibility** |  |  |  |  |  | 51.039 | 8 | **<.001** |
| Separated from partner/spouse | 107 (25.1) | 66 (15.5) | 48 (11.2) | 41 (9.6) | 165 (38.6) |  |  |  |
| Separated from immediate family | 283 (41) | 87 (12.6) | 93 (13.5) | 75 (10.9) | 152 (22) |  |  |  |
| Temporary visa holders | 83 (33.7) | 35 (14.2) | 32 (13) | 17 (6.9) | 79 (32.1) |  |  |  |
| **Gender (n=1350)** |  |  |  |  |  | 7.414 | 4 | **.116** |
| Female | 333 (33.2) | 135 (13.5) | 136 (13.6) | 103 (10.3) | 295 (29.4) |  |  |  |
| Male | 137 (39.4) | 53 (15.2) | 35 (10.1) | 29 (8.3) | 94 (27) |  |  |  |
| **Mental health services offered** |  |  |  |  |  | 13.484 | 4 | **.009** |
| Yes | 73 (27.4) | 37 (13.9) | 37 (13.9) | 21 (7.9) | 98 (36.8) |  |  |  |
| No | 400 (36.5) | 151 (13.8) | 136 (12.4) | 112 (10.2) | 298 (27.2) |  |  |  |
| **Medical advice offered** |  |  |  |  |  | 34.718 | 4 | **<.001** |
| Yes | 44 (22.6) | 19 (9.7) | 27 (13.8) | 16 (8.2) | 89 (45.6) |  |  |  |
| No | 429 (36.7) | 169 (14.5) | 146 (12.5) | 117 (10) | 307 (26.3) |  |  |  |
| **Government financial assistance offered** |  |  |  |  |  | 15.424 | 4 | **.004** |
| Yes | 25 (20.2) | 16 (12.9) | 19 (15.3) | 14 (11.3) | 50 (40.3) |  |  |  |
| No | 448 (36.2) | 172 (13.9) | 154 (12.4) | 119 (9.6) | 346 (27.9) |  |  |  |
| **Traveler registration offered** |  |  |  |  |  | 2.313 | 4 | .678 |
| Yes | 85 (36.8) | 34 (14.7) | 24 (10.4) | 25 (10.8) | 63 (27.3) |  |  |  |
| No | 388 (34.3) | 154 (13.6) | 149 (13.2) | 108 (9.5) | 333 (29.4) |  |  |  |
| **Emergency accommodation offered** |  |  |  |  |  | 3.602 | 4 | .476 ^a^ |
| Yes | 5 (26.3) | 3 (15.8) | 3 (15.8) | 0 | 8 (42.1) |  |  |  |
| No | 468 (34.8) | 185 (13.8) | 170 (12.6) | 133 (9.9) | 296 (29.1) |  |  |  |
| **Financial stress** |  |  |  |  |  | 85.881 | 4 | **<.001** |
| Yes | 275 (28.3) | 134 (13.8) | 117 (12) | 105 (10.8) | 340 (35) |  |  |  |
| No | 198 (50.5) | 54 (13.8) | 56 (14.3) | 28 (7.1) | 56 (14.3) |  |  |  |
| **Homelessness** |  |  |  |  |  | 29.403 | 4 | **<.001** |
| Yes | 27 (21.1) | 13 (10.2) | 13 (10.2) | 12 (9.4) | 63 (49.2) |  |  |  |
| No | 446 (36.1) | 175 (14.2) | 160 (13) | 121 (9.8) | 333 (27) |  |  |  |
| **Employment change** |  |  |  |  |  | 10.349 | 4 | **.035** |
| Yes | 217 (31) | 105 (15) | 89 (12.7) | 68 (9.7) | 221 (31.6) |  |  |  |
| No | 256 (38.6) | 83 (12.5) | 84 (12.7) | 65 (9.8) | 175 (26.4) |  |  |  |
| **Children** |  |  |  |  |  | 32.315 | 4 | **<.001** |
| Yes | 230 (42.6) | 81 (15) | 57 (10.6) | 48 (8.9) | 124 (23) |  |  |  |
| No | 243 (29.5) | 107 (13) | 116 (14.1) | 85 (10.3) | 272 (33) |  |  |  |
| **Employment during COVID-19** |  |  |  |  |  | 55.854 | 24 | **<.001** |
| Healthcare worker | 53 (39.3) | 25 (18.5) | 14 (10.4) | 13 (9.6) | 30 (22.2) |  |  |  |
| Government worker | 21 (31.3) | 10 (14.9) | 11 (16.4) | 6 (9) | 19 (38.4) |  |  |  |
| Essential services worker | 45 (25.4) | 21 (11.9) | 22 (12.4) | 31 (17.5) | 58 (32.8) |  |  |  |
| Educator | 41 (30.6) | 24 (17.9) | 22 (16.4) | 15 (11.2) | 32 (23.9) |  |  |  |
| Other worker | 233 (37.2) | 86 (13.7) | 78 (12.5) | 57 (9.1) | 172 (27.5) |  |  |  |
| Not in paid work | 62 (32.1) | 21 (10.9) | 24 (12.4) | 8 (4.1) | 78 (40.4) |  |  |  |
| Retired | 18 (58.1) | 1 (3.2) | 2 (6.5) | 3 (9.7) | 7 (22.6) |  |  |  |
| **Chronic Illness** |  |  |  |  |  | 15.312 | 8 | **.053** |
| Yes | 35 (23.8) | 22 (15) | 22 (15) | 16 (10.9) | 52 (35.4) |  |  |  |
| No | 430 (36.6) | 159 (13.5) | 146 (12.4) | 112 (9.5) | 327 (27.9) |  |  |  |
| Unsure | 8 (19) | 7 (16.7) | 5 (11.9) | 5 (11.9) | 17 (40.5) |  |  |  |
|  | | | | | | **F** | **df** | ***p*** |
| **Expenditure** |  |  |  |  |  | 4.052 | 4, 1358 | **.003** |
| **Age** |  |  |  |  |  | 15.912 | 4, 1358 | **<.001** |
| **Time separated or awaiting immigration** |  |  |  |  |  | .617 | 4, 1358 | .650 |
|  | **Stress** | | | | | **χ 2** | **df** | ***p*** |
| **Eligibility** |  |  |  |  |  | 44.425 | 8 | **<.001** |
| Separated from partner/spouse | 83 (19.4) | 40 (9.4) | 73 (17.1) | 109 (25.5) | 122 (29.6) |  |  |  |
| Separated from immediate family | 212 (30.7) | 72 (10.4) | 151 (21.9) | 139 (20.1) | 116 (16.8) |  |  |  |
| Temporary visa holders | 76 (30.9) | 27 (11) | 31 (12.6) | 53 (21.5) | 59 (24) |  |  |  |
| **Gender (n=1350)** |  |  |  |  |  | 8.249 | 4 | **.083** |
| Female | 257 (25.6) | 98 (9.8) | 194 (19.4) | 229 (22.9) | 224 (22.4) |  |  |  |
| Male | 113 (32.5) | 40 (11.5) | 57 (16.4) | 68 (19.5) | 70 (10.1) |  |  |  |
| **Mental health services offered** |  |  |  |  |  | 21.096 | 4 | **<.001** |
| Yes | 44 (16.5) | 30 (11.3) | 60 (22.6) | 60 (22.6) | 72 (27.1) |  |  |  |
| No | 327 (29.8) | 109 (9.9) | 255 (18.7) | 301 (22.1) | 297 (21.8) |  |  |  |
| **Medical advice offered** |  |  |  |  |  | 19.675 | 4 | **<.001** |
| Yes | 33 (16.9) | 17 (8.7) | 34 (17.4) | 51 (26.2) | 60 (30.8) |  |  |  |
| No | 338 (28.9) | 122 (10.4) | 221 (18.9) | 250 (21.4) | 237 (20.3) |  |  |  |
| **Government financial assistance offered** |  |  |  |  |  | 7.454 | 4 | **.114** |
| Yes | 23 (18.5) | 11 (8.9) | 23 (18.5) | 32 (25.8) | 35 (28.2) |  |  |  |
| No | 348 (28.1) | 128 (10.3) | 232 (18.7) | 269 (21.7) | 262 (21.1) |  |  |  |
| **Traveler registration offered** |  |  |  |  |  | 3.001 | 4 | .558 |
| Yes | 62 (26.8) | 29 (12.6) | 41 (17.7) | 55 (23.8) | 44 (19) |  |  |  |
| No | 309 (27.3) | 110 (9.7) | 214 (18.9) | 246 (21.7) | 253 (22.3) |  |  |  |
| **Emergency accommodation offered** |  |  |  |  |  | 2.237 | 4 | .709 ^a^ |
| Yes | 6 (31.6) | 2 (10.5) | 3 (15.8) | 2 (10.5) | 6 (31.6) |  |  |  |
| No | 365 (27.2) | 137 (10.2) | 252 (18.8) | 299 (22.2) | 291 (21.7) |  |  |  |
| **Financial stress** |  |  |  |  |  | 69.389 | 4 | **<.001** |
| Yes | 215 (22.1) | 91 (9.4) | 176 (18.1) | 238 (24.5) | 251 (25.8) |  |  |  |
| No | 156 (39.8) | 48 (12.2) | 79 (20.2) | 63 (16.1) | 46 (11.7) |  |  |  |
| **Homelessness** |  |  |  |  |  | 18.470 | 4 | **<.001** |
| Yes | 27 (21.1) | 10 (7.8) | 13 (10.2) | 36 (28.1) | 42 (32.8) |  |  |  |
| No | 344 (27.9) | 129 (10.4) | 242 (19.6) | 265 (21.5) | 255 (20.6) |  |  |  |
| **Employment change** |  |  |  |  |  | 11.638 | 4 | **.020** |
| Yes | 172 (24.6) | 62 (8.9) | 133 (19) | 162 (23.1) | 171 (24.4) |  |  |  |
| No | 199 (30) | 77 (11.6) | 122 (18.4) | 139 (21) | 126 (19) |  |  |  |
| **Children** |  |  |  |  |  | 34.954 | 4 | **<.001** |
| Yes | 180 (33.3) | 66 (12.2) | 108 (20) | 96 (17.8) | 90 (16.7) |  |  |  |
| No | 191 (23.2) | 73 (8.9) | 147 (17.9) | 205 (24.9) | 207 (25.2) |  |  |  |
| **Employment during COVID-19** |  |  |  |  |  | 48.540 | 24 | **.002** |
| Healthcare worker | 43 (31.9) | 17 (12.6) | 26 (19.3) | 26 (19.3) | 23 (17) |  |  |  |
| Government worker | 15 (22.4) | 11 (16.4) | 13 (19.4) | 16 (23.9) | 12 (17.9) |  |  |  |
| Essential services worker | 37 (20.9) | 15 (8.5) | 32 (18.1) | 52 (29.4) | 41 (23.2) |  |  |  |
| Educator | 37 (27.6) | 15 (11.2) | 21 (15.7) | 36 (26.9) | 25 (18.7) |  |  |  |
| Other worker | 173 (27.6) | 70 (11.2) | 127 (20.3) | 125 (20) | 131 (20.9) |  |  |  |
| Not in paid work | 49 (25.4) | 11 (5.7) | 30 (15.5) | 42 (21.8) | 61 (31.6) |  |  |  |
| Retired | 17 (54.8) | 0 | 6 (19.4) | 4 (12.9) | 4 (12.9) |  |  |  |
| **Chronic Illness** |  |  |  |  |  | 18.551 | 8 | **.017** |
| Yes | 28 (19) | 9 (6.1) | 33 (22.4) | 32 (21.8) | 45 (30.6) |  |  |  |
| No | 336 (28.6) | 125 (10.6) | 213 (18.1) | 261 (22.2) | 239 (20.4) |  |  |  |
| Unsure | 7 (16.7) | 5 (11.9) | 9 (21.4) | 8 (19) | 13 (31) |  |  |  |
|  | | | | | | **F** | **df** | ***p*** |
| **Expenditure** |  |  |  |  |  | 1.830 | 4, 1358 | **.121** |
| **Age** |  |  |  |  |  | 14.338 | 4, 1358 | **<.001** |
| **Time separated or awaiting immigration** |  |  |  |  |  | .656 | 4, 1358 | .623 |

1. Fischer’s exact test

**χ 2 , chi squared; df, degrees of freedom; F, analysis of variance;*p* , probability value (statistically significant <.2).**
