# Supplementary material for: Psychological and financial impacts of COVID-19-related travel measures: An international cross-sectional study
Source: PLoS One. 2022 Aug 5;17(8):e0271894. doi: 10.1371/journal.pone.0271894 (PMC9355190; doi:10.1371/journal.pone.0271894)
Supplement: Attachment — Submitted filename: Response to Reviewers.docx [file pone.0271894.s002.docx]

**Journal Requirements**

- *Reference for the initial study has been updated with official publication citation (16).*

**Editor comments**

Please improve methods with reference to standard process of conducting survey based studies e.g. CHERRIES checklist, 10.3346/jkms.2020.35.e398

- *Added missing ‘Cherries checklist’ details in methods related to – Sampling design, response rate diagram and handling of incomplete questions.*

**Reviewer comments**

*Reviewer 2*

1. "Using a key, the resulting scores are categorised as normal,
   mild, moderate, severe, or extremely severe depression, anxiety, or stress (DAS)." Relevant reference is required; please also state the cut-offs.
   a. *A table has been created to reflect cut off scores and a reference has been added*
2. "Only completed surveys were analysed." There are ways of dealing with missing data.
   a. *Of the respondents who did complete questions on the outcome (DAS score), most did not complete the last page of the survey which contained all questions related to demographic information. We considered the use of multiple imputation however these methods are best suited when missing information is sparse. In our scenario, participants were missing all demographic information. We have compared the outcomes for those with and without missing demographic information and found the distribution of scores to be comparable. Therefore, we do not have a reason to believe that there are meaningful differences between the those with and without missing demographic information and are treating them as missing completely at random (Methods under “Statistical Analysis” has been updated to reflect this). A third group of respondents began the survey but did not complete questions related to outcome. As imputation methods are best used for predictor variables, we did not include any of these responders in the analysis. A new diagram has been created to reflect the breakdown of completed responses - Figure 1. Flowchart of the participant inclusion process (2).tiff.*
3. "Chi-square analyses " which chi-squared test was used?
   a. *Sentence has been updated to reflect the use of chi square test for associations*
4. How was the normality of data checked?
   a. *Continuous outcomes were visually assessed for normality. Methods has been updated to reflect this “No errors, influential outliers or multicollinearity amongst variables was identified. Diagnostics confirmed model assumptions were met.”*
5. Manufacturer and country of origin should be given for statistical software.
   a. *Citation for SPSS guided by: https://www.ibm.com/support/pages/how-cite-ibm-spss-statistics-or-earlier-versions-spss*
6. Which program was used for online survey?
   a. *Discussed and referenced in ‘Study design and study population’ section - “The online open survey was developed using survey tool Qualtrics”*
7. "Statistical significance was p > 0.05." Please recheck.
   a. *Thank you for picking this up. The sentence has been corrected to p < 0.05*
8. How was the sample size arrived at?
   a. *A minimum sample size of 385 calculated to allow for a proportion of 50% reporting moderate to severe DAS scores with a margin of error of 5%*
9. What was the response rate?
   a. *Due to the study design using opportunistic sampling, the response rate cannot be determined.*
10. Any statistical correction for multiple comparisons?
    a. *The results of the regression analyses are considered to be hypothesis generating, therefore corrections for multiple comparisons have not been applied.*

*Reviewer 3*

The process of selection of the study participants was not very clear. Authors spoke about a previous study but the current method of selection could be explained better.
Page 3 last paragraph- 2nd line - " forced separation" instead of " forced separated".
Could the bias of relatively high number of female respondents be resolved by using weighted values?

- *The opportunistic sampling strategy precludes the identification of the sampling frame, therefore we are unable to apply weighting methods. We have accounted for potential confounding by the inclusion of sex and other important demographic variables in the regression models.*
